# 

**Published:** 1916-02

**Authors:** 


### Publishers' Special Announcements

There is something splendid about the work that has been accomplished by the dental profession and there are still splendid, huge tasks, for it to accomplish.

It is comparatively easy nowadays to learn the profession of dentistry compared to what it was in the early days when the Dental Register began its work of dental journalism. In those days the formulas that are now in common use were being worked out and the methods of dentistry as now practiced were being reduced to the procedure now followed. It is easier because the way has been paved by the pioneers whose practices have been tested by time. Men who devoted their lives to the work of dentistry and gave their experience to the profession have a place in the hall of fame and the names of the contemporary devotees will be there also.

The Dental Register has devoted much space to the learned heads of the profession during its seventy years of work and is doing its share in the promulgation of dental knowledge today. Conservatism and truth are the byewords of both its written articles and its advertisements and the dental profession knows it can rely and depend upon the Dental Register.

In the great work to come the dental profession is assured that the Dental Register will always lend a helping hand and work for the highest and best interests of the dentists.

---

**HAVE YOU MONEY THAT IS LOAFING; WHY NOT PUT IT TO WORK?**—By opening a deposit account with us. Deposit $95.00 and receive credit for $100.00 against which you can purchase all supplies including precious metals when bought at pennyweight rates from THE SAM'L A. CROCKER CO.

---

Subscriptions to the Dental Register will be received at office of The Sam'l A. Crocker Co. Price per year $1.00. Dental Dealers throughout the United States receive subscriptions for The Dental Register. Send your orders for balance of 1915 and 1916 through your dealer or direct. Founded 1846, now in 69th year. The Dental Register is the most reliable and conservative Dental Magazine published. Send in your orders now.

### ANNOUNCEMENTS

FREDERIC WARD PUTNAM. It would be difficult in a few words to do justice to the memory of Professor Frederic Ward Putnam who died on the fourteenth of last August at the age of seventy-six. Best known as the curator of the Peabody Museum of Harvard University from 1875 to 1909, and leaving in the work accomplished in that department of the University his most fitting monument, he was also vitally connected with numerous other institutions and scientific bodies and was a member of a large number of scientific societies in America and abroad. His own estimate of his life work made his best accomplishment to consist in "the establishing and development of new departments of anthropology in Harvard and California Universities; the inception and development of anthropological museums; and the study and preservation of pre-historic monuments in the United States." He wrote more than four hundred papers, reports and notes on zoology and anthropology besides a large amount of editorial work. The value of his activities was everywhere recognized. The French government conferred upon him the Cross of the Legion of Honor in 1896 and he received the Drexel Gold Medal from the University of Pennsylvania in 1903; both these in recognition of his services in aid of American archæology.

"As a boy he was a helper about home, worked with his father in cultivating and propagating plants, and he considered that this early training in work and in regular duties had much to do with making him handy in the use of tools and ready in the emergencies of life." He had a passion for facts, but he was also an inspirer of students seeking facts, and in this particular his success as an instructor was signal.

His relation with the American Academy of Dental Science arose doubtless out of his catholicity of mind and his interest in every department of anthropology. It is a privilege to review, even so briefly as this, and to hold in memory the life of a man so devoted to duty, so direct in his aims, so thorough in his methods, so rich in experience and so widely acclaimed for the valuable result of his labors.

HENRY H. PIPER,
WILLIAM P. COOKE,
AMOS I. HADLEY.

---

EXHIBIT OF DENTAL MANUFACTURERS CLUB. An exhibit will be held in the spacious and commodious Hotel Auditorium, Chicago, Illinois, on April 4th, 5th, 6th and 7th, 1916. The Auditorium Hotel will be the scene of one of the greatest exhibits of Dental Manufacturers, including equipment, furniture, materials and instruments ever shown in this country.

Preparations are under way for an elaborate, scientific and most highly interesting and educational exhibit, with table clinics, and demonstration under direction of men "who make the goods," assisted by expert demonstrators and lecturers.

Parking space for automobiles will be arranged for, as will all other details for the comfort and convenience of visitors.

The Exhibit Committee is endeavoring to make this exhibit the greatest of all exhibits of dental manufacturers and most highly interesting and educational in the way of sound and practical table demonstrations of particular interest to the profession.

---

NORTHERN OHIO DENTAL ASSOCIATION. The annual session of the Northern Ohio Dental Association will be held in Cleveland, June 1, 2, 3, 1916.

Sandusky, Ohio. CLARENCE D. PECK, *Secy.*

